# Brazilian Semi-Arid Mangroves-Associated Microbiome as Pools of Richness and Complexity in a Changing World

**DOI:** 10.3389/fmicb.2021.715991

**Published:** 2021-08-26

**Authors:** Tallita Cruz Lopes Tavares, Walderly Melgaço Bezerra, Leonardo Ribeiro Oliveira Normando, Alexandre Soares Rosado, Vânia Maria Maciel Melo

**Affiliations:** ^1^Marine Sciences Institute, Federal University of Ceará (UFC), Fortaleza, Brazil; ^2^Laboratory of Microbial Ecology and Biotechnology, Department of Biology, Federal University of Ceará (UFC), Fortaleza, Brazil; ^3^Biological and Environmental Science and Engineering Division, King Abdullah University of Science and Technology (KAUST), Thuwal, Saudi Arabia

**Keywords:** soil microbial community, *Rhizophora mangle*, red mangrove, blue carbon, amplicon sequence variants, network co-occurrence analysis

## Abstract

Mangrove microbiomes play an essential role in the fate of mangroves in our changing planet, but the factors regulating the biogeographical distribution of mangrove microbial communities remain essentially vague. This paper contributes to our understanding of mangrove microbiomes distributed along three biogeographical provinces and ecoregions, covering the exuberant mangroves of Amazonia ecoregion (North Brazil Shelf) as well as mangroves located in the southern limit of distribution (Southeastern ecoregion, Warm Temperate Southwestern Atlantic) and mangroves localized on the drier semi-arid coast (Northeastern ecoregion, Tropical Southwestern Atlantic), two important ecotones where poleward and landward shifts, respectively, are expected to occur related to climate change. This study compared the microbiomes associated with the conspicuous red mangrove (*Rhizophora mangle*) root soils encompassing soil properties, latitudinal factors, and amplicon sequence variants of 105 samples. We demonstrated that, although the northern and southern sites are over 4,000 km apart, and despite *R. mangle* genetic divergences between north and south populations, their microbiomes resemble each other more than the northern and northeastern neighbors. In addition, the northeastern semi-arid microbiomes were more diverse and displayed a higher level of complexity than the northern and southern ones. This finding may reflect the endurance of the northeast microbial communities tailored to deal with the stressful conditions of semi-aridity and may play a role in the resistance and growing landward expansion observed in such mangroves. Minimum temperature, precipitation, organic carbon, and potential evapotranspiration were the main microbiota variation drivers and should be considered in mangrove conservation and recovery strategies in the Anthropocene. In the face of changes in climate, land cover, biodiversity, and chemical composition, the richness and complexity harbored by semi-arid mangrove microbiomes may hold the key to mangrove adaptability in our changing planet.

## Graphical Abstract

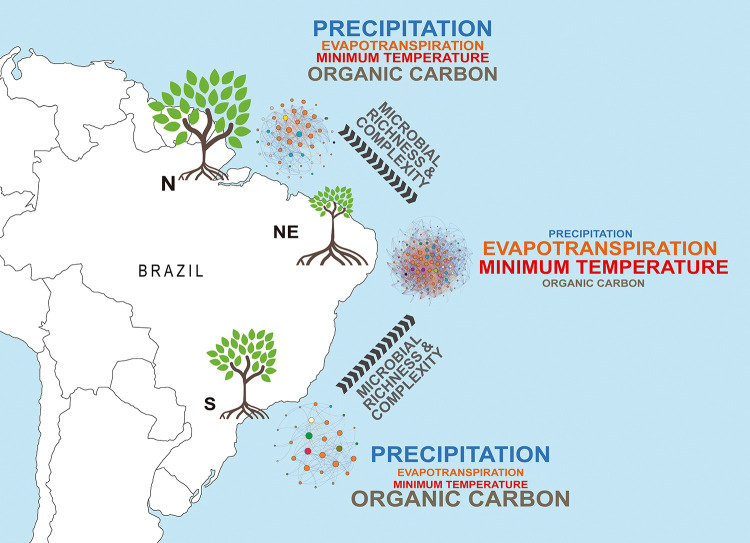

## Introduction

Mangroves cover 75% of tropical and subtropical coastlines (Giri et al., 2011), comprising 137,600 km^2^ distributed throughout 118 countries. Due to their high primary productivity (218 ± 72 Tg C yr^–1^) (Bouillon et al., 2008) and high carbon-storage capacity (24 Tg C yr^–1^) (Alongi, 2014), mangroves can play a crucial role as blue-carbon sinks (Mcleod et al., 2011), provide valuable ecosystems services (Barbier et al., 2011) and support adaptation to climate change (Lovelock and Duarte, 2019). However, when disturbed through land-use changes, mangroves can become a source of large quantities of greenhouse gases (GHG) (Kauffman et al., 2020). For instance, mangrove conversion to agriculture or aquaculture can result in greenhouse gas emissions of 1,067–3,003 Mg CO_2_e ha^–1^, most of this emissions originated from soil carbon pool losses (Kauffman et al., 2018a,b). Indeed, under “business as usual” scenario rates of mangrove loss, emissions could reach 3,394 Tg CO_2__*eq*_ when considering foregone soil carbon sequestration (Adame et al., 2021). Microbes are the main players that contribute to these and other mangrove ecosystem services (Allard et al., 2020). They constitute the mangrove microbiome, which comprises taxonomically and functionally diverse microorganisms that participate in element cycling, organic matter decomposition and mineralization (Ochoa-Gómez et al., 2019), and promote plant growth (Tong et al., 2019), being directly or indirectly related to mangrove ecosystems services.

Forces shaping soil microbial dynamics have become recently well understood (Fierer, 2017). However, this knowledge can be helpful to understand how microbes mediate ecosystem functioning and improve mangrove conservation and management (Tong et al., 2019). Several factors have been reported as drivers of the distribution and structure of bacterial communities of mangrove soils, such as the presence or absence of vegetation (Jiang et al., 2013; Gomes et al., 2014; Prakash et al., 2015; Chen et al., 2016; Liu et al., 2017), depth (Taketani et al., 2010; Andrade et al., 2012; Otero et al., 2014; Basak et al., 2016; Luis et al., 2019), human activities (Dias et al., 2011; Peixoto et al., 2011; Fernandes et al., 2014; Nogueira et al., 2015; Basak et al., 2016; Erazo and Bowman, 2021), tidal cycles (Zhang et al., 2018), and physicochemical properties (Mendes et al., 2012; Colares and Melo, 2013; Mendes and Tsai, 2014, 2018; Chen et al., 2016; Zhu et al., 2018). However, many of them cover different areas of a certain mangrove or compare pristine versus impacted ecosystems, demonstrating microbial shifts associated with preservation status in a local scale. In contrast, few researchers have considered microbial communities on a latitudinal scale (Li et al., 2021; Zhang et al., 2021). Consequently, the factors regulating the biogeographical distribution of mangrove microbial communities remain understudied.

In Brazil, mangroves cover 9,900 km^2^ (Diniz et al., 2019), the third-largest worldwide mangrove area in a single country (8.5% of the global mangrove area) (Giri et al., 2011), but also face the fourth highest potential annual CO_2_ emission due to deforestation (Atwood et al., 2017). Brazilian mangroves extend from the far-northern Brazilian coast (04°20′N) to the southern coast (28°30′S) (Schaeffer-Novelli et al., 2000) in a patchy distribution (Pil et al., 2011) where mangrove tree species, such as the conspicuous *Rhizophora mangle* (red mangrove), display a genetic subdivision between northern and southern populations, linked to climatic and oceanographic characteristics (Francisco et al., 2018). In general, mangroves are more abundant, diverse, and productive in humid climates, while freezing temperatures can lead to mortality, loss of above-ground biomass, and reduced productivity and reproduction. Mangrove forests are also sensitive to hypersaline conditions; prevalent in low precipitation areas displaying reduced freshwater inputs (Lovelock et al., 2016), which can limit the distribution and diversity of mangroves on arid and semi-arid coasts (Osland et al., 2018). Still, ecosystem carbon stocks of wet Amazon mangroves and dry semi-arid Brazilian Northeast were not significantly different, with soils and roots comprising ∼69% of the total ecosystem carbon stocks (Kauffman et al., 2018b). Microbial communities play crucial roles in mangrove biogeochemistry, nutrient cycling, and plant productivity (Allard et al., 2020), however, knowledge on how mangrove microbiota act in contrasting conditions remains sparse. In addition, there are no studies on the role of microorganisms on the processes of expansion or retraction of mangrove forests linked to sea level rise (Ward and Lacerda, 2021). Thus, considering mangroves as climate change sentinels (Alongi, 2015), understanding the role of microorganisms in their stability can provide crucial information to protect and restore them in such a changing world.

In this study, we examined the biogeographical distribution of the prokaryotic community associated with the roots of *R. mangle* populations, a widespread dominant mangrove tree species in the Atlantic and Eastern Pacific biogeographic regions, whose forests display high soil carbon stocks (Atwood et al., 2017). We hypothesized that such microbial communities would differ along the latitudinal transect, with increasing differences upon distance. We tested this hypothesis on soil samples collected from *R. mangle* root zones from seven Brazilian mangroves concentrated in three regions: north (Amazonia ecoregion), northeast (northeastern Brazil ecoregion), and south (southeastern Brazil ecoregion) (Spalding et al., 2007). We evaluated the diversity and composition of these microbial communities, explored the influence of soil physical and chemical properties as well as latitudinal factors, and examined co-occurrence patterns in the microbial communities based on 16S rDNA amplicons. Considering the vast extent of Brazilian mangroves, their potential value for climate mitigation, the role of microbial processes, and the possibilities of developing microbial-based interventions for monitoring and ecosystem rehabilitation, this study is a timely opportunity to advance in the understanding of the responses of mangroves and associated microbiomes to climate change.

## Materials and Methods

### Study Sites and Sampling

**FIGURE 1:**
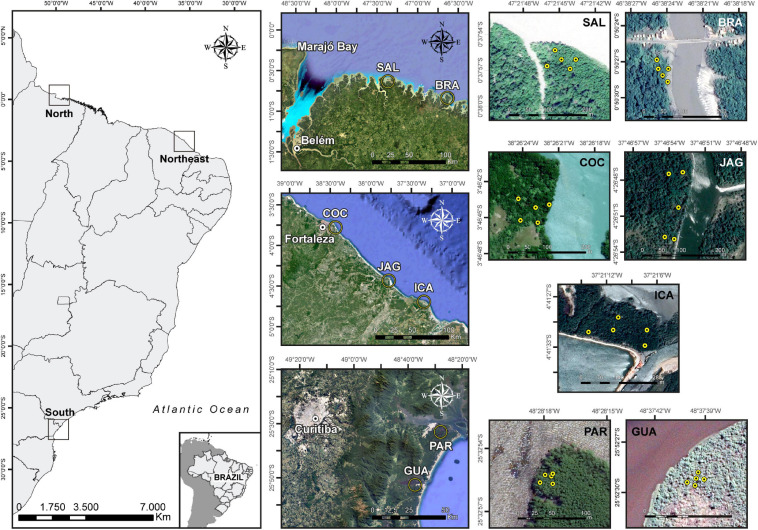
Map indicating the geographical locations of the sampling sites. Five sampling points were sampled at each mangrove, each comprising three subsamples. A total of seven mangroves were studied; in the North: Salinópolis (SAL) and Bragança (BRA); in the Northeast: Cocó (COC), Jaguaribe (JAG), and Icapuí (ICA); and in the South: Paranaguá (PAR) and Guaratuba (GUA).

The dataset consisted of 105 soil samples collected across northern, northeastern and southern Brazil: two northern (Bragança–BRA-N, 00°50′28.1″S 46°38′24.4″W; and Salinópolis–SAL-N, 00°37′56.1″S 47°21′44.8″W), three northeastern (Cocó–COC-NE, 03°46′44.2″S 38°26′22.9″W; Jaguaribe–JAG-NE, 04°26′50.3″S 37°46′53.2″W; and Icapuí–ICA-NE, 04°41′31.0″S 37°21′11.2″W), and two southern (Paranaguá–PAR-S, 25°32′55.3″S 48°28′17.6″W; and Guaratuba–GUA-S, 25°52′29.2″S 48°37′39.1″W) mangroves (Figure 1). The physiographic characteristics of Brazilian mangroves (Schaeffer-Novelli et al., 1990, 2000) and the regionalization of Brazilian coastline regarding bioclimatic factors (Ximenes et al., 2016) were considered in the soil sampling delineation in order to cover different habitats for *R. mangle*.

Five triplicate samples were obtained at each mangrove from *R. mangle*-vegetated root zones, totaling 15 samples per mangrove. Each of the five points was between 10 to 50 m apart. Soil samples were collected from the top 20 cm, with the uppermost 1 mm eliminated, using a soil core sampler of 10 cm in diameter and 1.5 L of volume. Sampling was done during low tide (max. tidal amplitude of 0.2 m) between August and November 2013. From the collected samples, an equivalent of 50 mg of soil were destined to DNA extraction, which were kept on ice during transport and stored at −20°C until processing. The remaining soil was destined to physical and chemical characterizations and was maintained at room temperature until analysis. All the 105 soil samples were used for physical and chemical characterization as well as for DNA sequencing.

### Physical and Chemical Characterization

Soil physical and chemical properties were determined in triplicate for all samples, using standard laboratory protocols, except for salinity, which was measured *in situ*. Soil pH was determined in a 1:2.5 soil/water extract. Particle size was analyzed using the pipette method (Teixeira et al., 2017). Organic-matter and organic-carbon contents were determined by the loss-by-ignition method (Wright et al., 2008; Schulte and Hopkins, 2015). Total nitrogen was measured by the micro-Kjeldahl method with adaptations (Baethgen and Alley, 1989). Data on annual accumulated precipitation, average annual potential evapotranspiration, average annual maximum and minimum temperatures, and average annual humidity were obtained from the HidroWeb and INMET (Brazilian National Institute of Meteorology) databases.

### DNA Extraction and Library Preparation

Total DNA was extracted using the PowerLyzer PowerSoil DNA Isolation Kit (MO BIO Laboratories, Carlsbad, CA, United States), following the manufacturer’s instructions. DNA quality was verified with a NanoDrop ND-1000 spectrophotometer (Thermo Scientific, Waltham, MA, United States). The V4 region of 16S rRNA gene was targeted and amplified using the 515F/806R primer set (Caporaso et al., 2011), with barcodes in the forward primer using the following program: 95°C for 4 min, 60°C for 1 min, 72°C for 2 min, followed by 25 cycles at 94°C for 1 min, 60°C for 1 min, and 72°C for 2 min. PCR products were purified using calibrated AMPure XP beads (Beckman Coulter, Indianapolis, IN, United States), and paired-end sequenced using an Illumina MiSeq Reagent Kit v2 (500 cycles, 2 × 250 bp) on an Illumina MiSeq sequencer (Illumina, San Diego, CA, United States) at the Genomic and Bioinformatic Facility (CEGENBIO) of the Drug Research and Development Center (NPDM), belonging to the Federal University of Ceará.

### Sequence Data Processing

After sequencing, Illumina adapter sequences were trimmed from already-demultiplexed raw fastq files using Cutadapt v1.8 (Martin, 2011) in paired-end mode, and the reads quality was assessed using FastQC v.0.11.8 (Andrews, 2012) and vsearch v2.10.4 (Rognes et al., 2016). Subsequent analyses were performed in the R v3.5.3 environment (R Core Team, 2016), following the DADA2 v1.11.1 package (Callahan et al., 2016) pipeline for obtaining a table of non-chimeric amplicon sequence variants (ASVs) free of low-quality and non-prokaryotic sequences (ASVs; sequences differing by as little as one nucleotide) (Callahan et al., 2017). Taxonomy assignment and removal of non-prokaryotic sequences was performed against the SILVA reference database (release 132) (Yilmaz et al., 2014). The 16S rRNA data were deposited in the NCBI Bioproject database with accession no. PRJNA283936^1^.

### Data Analyses

Environmental data were analyzed by a principal component analysis (PCA), with measurements transformed to log (x + 1), except for pH. Alpha-diversity estimators were calculated and tested for normality by the Shapiro–Wilk test. As the Shannon diversity and Simpson evenness were parametric, a one-way analysis of variance and Tukey’s honestly significant difference (HSD) *post hoc* tests were used for multiple comparisons of means at a 95% confidence interval. For Chao1 and Observed ASVs, we used the Kruskal–Wallis non-parametric test.

The response patterns of alpha-diversity to the abiotic driving factors were evaluated using simple linear models. Linear regressions, coefficients of determination (R^2^), and significance (*P*) were calculated in R environment using R base *stats* package. Indicator analysis was performed using the package *indicspecies* (de Cáceres et al., 2011).

Canonical correspondence analysis (CCA) was used to visualize the differences in community structure among the different mangroves and determine its correlation with environmental parameters, using Hellinger-transformed abundance matrix at ASV level and rarefied data. For this, biotic and abiotic matrices were previously analyzed using the detrended correspondence analysis (DCA) to evaluate the gradient size of ASV distribution, which indicated both linear and unimodal methods as suitable (length of Axis 1 = 3.0064). Subsequently, a preliminary CCA was performed with all available physical and chemical parameters. For variable reduction and creation of an efficient model from the most significant explanatory variables, forward selection of constraints using the VEGAN *ordistep* function (Oksanen et al., 2019) was performed, and only significant variables (*P* < 0.05) were retained in the second CCA. In order to avoid the horseshoe and arch effects, detrending (DCA) was initially used. It was followed by external linear constraints (CCA), which are suitable for preserving true arches (Palmer, 1993). The SIMPER (similarity percentage) analysis was used to identify the taxa primarily responsible for the differences observed in the ordination using seq-scripts release v. 1.0. (Steinberger, 2020). All plots were generated using the R v3.5.3 environment (R Core Team, 2016).

Functional analysis was performed using predict metagenomic functional content on PICRUSt2 (Phylogenetic Investigation of Communities by Reconstruction of Unobserved States, version 2.3.0.) with default settings (Douglas et al., 2020). The predicted genes were classified by alignment to KEGG Orthology (KO) database. The obtained gene predicted abundances were normalized by 16S rRNA gene copy number, and then used to predict the metagenomic functional content.

### Network Co-occurrence Analysis

A network analysis was performed to assess the complexity of interactions among microbial taxa. Non-random co-occurrence analyses were performed using FastSpar (Watts et al., 2019). For each network, *P*-values were obtained by 99 permutations of random selections of the data table, subjected to the same analytical pipeline. Statistically significant (*P* < 0.01) FastSpar correlations with a magnitude of 0.7 or −0.7 were included into the network analyses. The nodes in the reconstructed network represent taxa at the ASV level, whereas the edges represent significantly positive or negative correlations between nodes. Network graphs were constructed based on a set of measurements, including the number of nodes, number of edges, modularity, number of communities, mean node connectivity, mean path length, diameter, and cumulative degree distribution. Visualization and property measurements were performed with the Gephi interactive platform (Bastian et al., 2009).

## Results

### Characterization of Abiotic Driving Factors

We analyzed 105 mangrove soil samples distributed along ∼4,000 km spanning three climatic zones: tropical rainforest (Af); hot semi-arid (BSh); and humid subtropical (Cfa) according to Köppen (1936). The mangroves were separated based on principal components into groups characterized by different environmental conditions.

**FIGURE 2:**
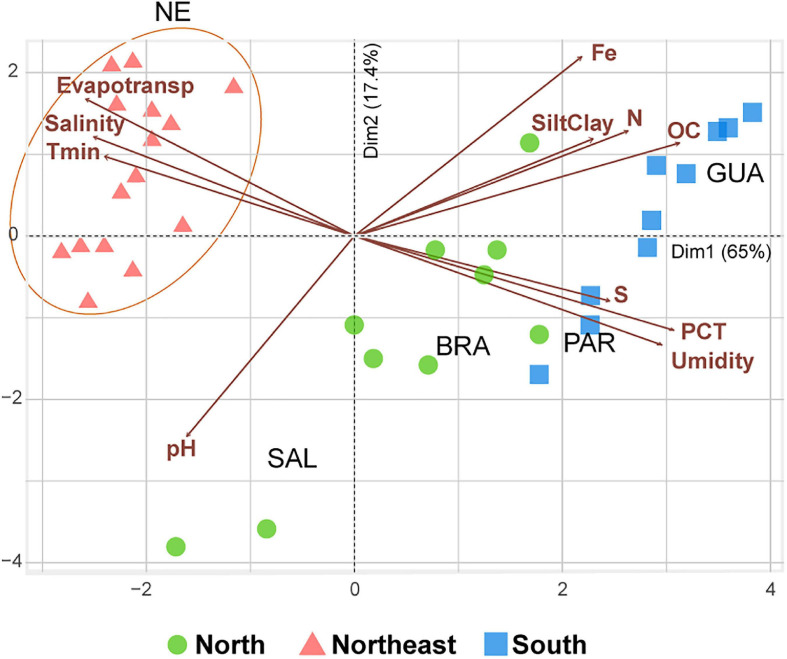
Triplot diagram of the Principal component analysis (PCA) indicating the orientation pattern based on the variability of abiotic variables in the seven studied mangroves. The two first principal components are plotted with the proportion of variance explained by each component shown in parentheses in the axis titles. Abbreviated abiotic variables: Tmin (Minimum Temperature); PCT (Precipitation); Evapotrans (Potential Evapotranspiration); Fe (iron); N (Total Nitrogen); OC (Organic Carbon); SiltClay (Silt and Clay content); and S (Sulfur).

Northeastern mangroves were correlated with elevated salinity, evapotranspiration, and minimum temperature as well as low sulfur, precipitation, and humidity. Elevated precipitation, pH, humidity, and sulfur content were the main components for northern mangroves, while southern samples were correlated with elevated precipitation, humidity, organic carbon, nitrogen, iron, sulfur, and silt-clay contents. Principal components 1 and 2 explained 82.4% of the dataset variability and provided a clear separation of Northeastern samples (Figure 2).

### Richness and Diversity Estimations

The 105-mangrove soil 16S rRNA libraries analyzed in the present work generated a total of 13,230,772 sequences, with 4,791,858 sequences remaining after processing (Supplementary Table 1). The dataset was rarefied to 47,707 based on the minimal sequence count per sample (Supplementary Figure 1), resulting in a normalized dataset comprising 16,104 ASVs, of which 14,992 and 1,112 were identified as Bacteria and Archaea, respectively. All samples reached Good’s coverage above 99% and rarefaction curves for the observed ASVs were very close to reaching the asymptotes, confirming that sequencing and sampling efforts sufficiently captured the diversity of taxa within samples (Supplementary Figure 2).

**FIGURE 3:**
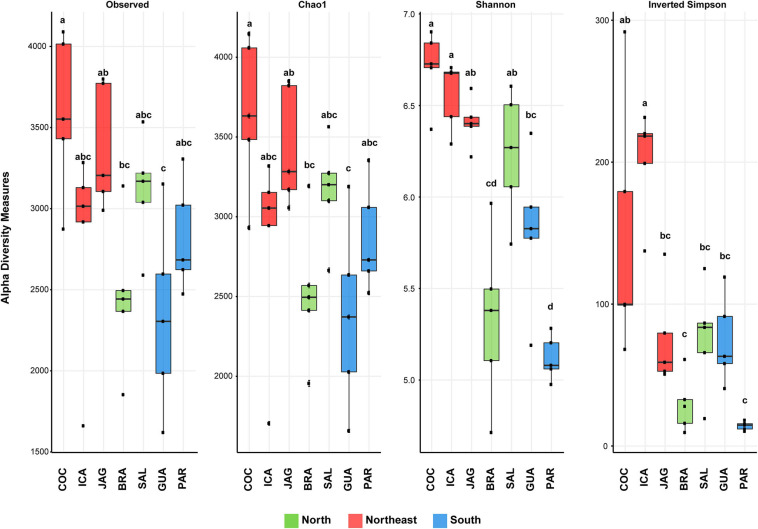
Box plots showing the richness (Observed richness and Chao1) and alpha diversity (Shannon and Inverted Simpson indices) of microbial communities in the root zone of *Rhizophora mangle* from seven mangroves in the north (green), northeast (red), and south (blue) Brazilian regions. Taxonomic diversity is based on ASV level. Error bars represent the standard deviation of 15 independent replicates (five sampling points with three replicates from each mangrove). Different lowercase letters refer to significant differences between treatments based on Tukey’s HSD test (*P* < 0.05) for the Shannon and Inverted Simpson indices and the Kruskal–Wallis test for Observed richness and Chao1.

The alpha diversity was highest in northeastern mangroves, as indicated by both the Shannon index and Simpson evenness, regardless of the dataset size. Interestingly, the southern mangrove PAR-S, which resulted in the highest number of sequences (Supplementary Figures 1, 2 and Supplementary Table 2), displayed the lowest Shannon and inverted Simpson indices, indicating that this microbial community is the least diverse, followed by southern GUA-S and northern BRA-N. COC-NE and ICA-NE displayed higher evenness, while JAG-NE did not possess as even a distribution as its counterparts in the northeast. Also, BRA-N and PAR-S exhibited a high dominance level (Figure 3).

**FIGURE 4:**
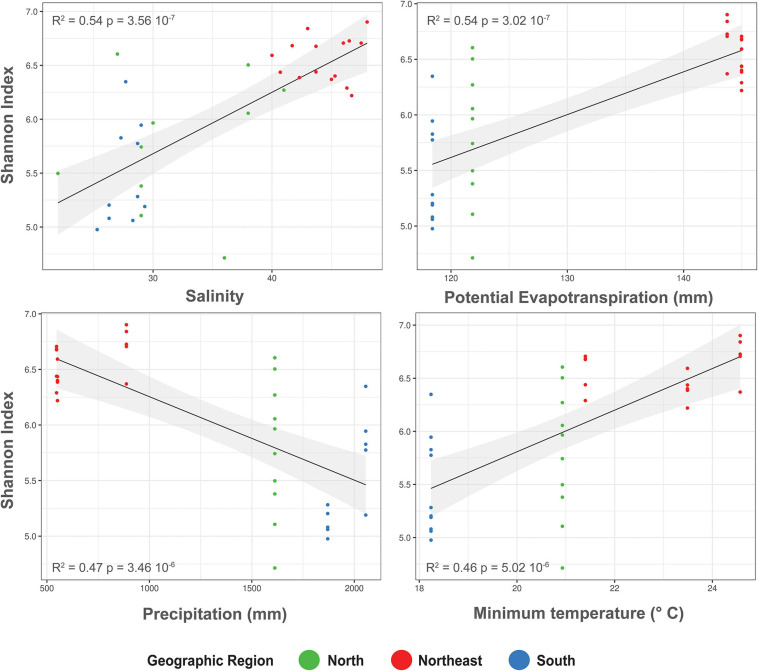
Relationships between Shannon index and Salinity, Potential Evapotranspiration, Precipitation, and Minimum temperature in the *Rhizophora mangle* root-associated soil microbiomes. The black lines represent linear regressions, and the shaded areas show the 95% confidence interval.

Furthermore, we explored the response patterns of alpha-diversity to the abiotic driving factors and found significant linear regressions with a potential tendency of higher alpha-diversity along with increased salinity, potential evapotranspiration, and minimum temperature as well as decreased alpha-diversity with increased precipitation (Figure 4). This contributed to separate northeastern mangrove samples from northern and southern ones.

### Microbial Community Composition

High-resolution community profiles were generated by processing reads using a denoised pipeline to resolve 16S rRNA gene ASV at the single-nucleotide level. Microbiomes’ members were classified into 63 phyla, 16 of which displaying abundances above 1%. Bacterial sequences were predominant in most samples (52–86%), while Archaea was present in elevated abundances (13–48%) only in some mangroves. The distribution of the dominant members displayed Deltaproteobacteria as the most prevalent class in most samples (30–40% in COC-NE and JAG-NE; 40–60% in BRA-N and SAL-N; and 55% in PAR-S), except for GUA-S and ICA-NE, where Thermoplasmata (Phylum Euryarchaeota) was dominant (∼40–50%). In all northern mangroves and PAR-S, Deltaproteobacteria still predominated but was followed by Thermoplasmata (20–25%). In northeastern mangroves, Gammaproteobacteria was the second more prevalent class in COC-NE and JAG-NE. Another notable composition feature was the presence of ∼7% of Campylobacteria (Phylum Epsilonbacteraeota) in northern mangroves. Other dominant phyla comprised Planctomycetes, Acidobacteria, Bacteroidetes, and Chloroflexi in all mangroves (Supplementary Figure 3).

**FIGURE 5:**
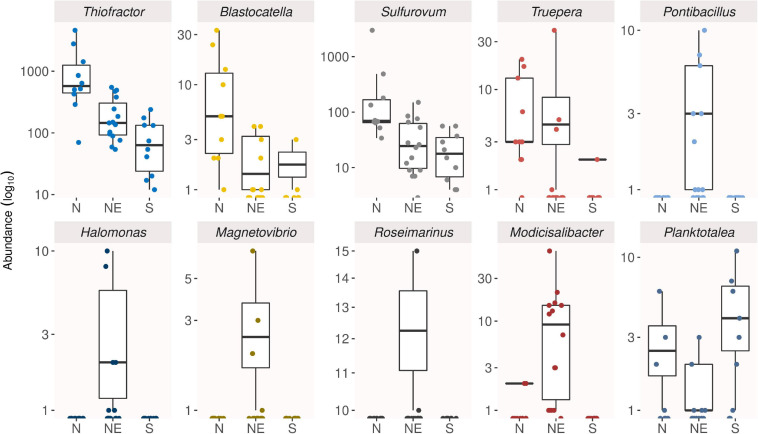
Box plots showing the abundance distribution of the main indicator genera of *Rhizophora mangle* root-associated soil microbiomes at North (N), Northeast (NE), and South (S).

Then, the indicator analysis revealed 33 out of 499 genera as the main responsible for differences among groups of samples reunited in three geographical regions: nine of them were associated with northern mangroves, 22 with northeastern mangroves, and two with southern ones (Supplementary Table 3). Most indicator genera were part of the rare biosphere, with abundance below 0.01%, and some of them were moderately halophilic groups and almost exclusive of mangroves from northeastern Brazil, such as *Pontibacillus*, *Halomonas*, *Magnetovibrio*, *Roseimarinus*, and *Modicisalibacter* (Figure 5).

### Main Environmental Drivers of *R. mangle* Microbial Community

Permutational multivariate analysis of variance (PERMANOVA) with 1,000 permutations was used to test for significant effects of geographical distance. We detected significant differences between north × northeast (*F* = 4.2028; *P* = 0.002), north × south (*F* = 6.0065; *P* = 0.001), and northeast × south (*F* = 6.4463; *P* = 0.001), which evidenced the greater differences between northeastern and southern mangroves. In order to consider the influence of the plant genotype on the microbial communities, we also tested the genetic differences between northern, northeastern, and southern *R. mangle* populations. Therefore, we used data on the number of effective alleles and observed heterozygosity extracted from Francisco et al. (2018). The PERMANOVA detected a significant relationship between the associated soil bacterial communities and both the number of effective alleles (*F* = 4.9676; *P* < 0.001) and the observed heterozygosity (*F* = 5.9111; *P* < 0.001).

**FIGURE 6:**
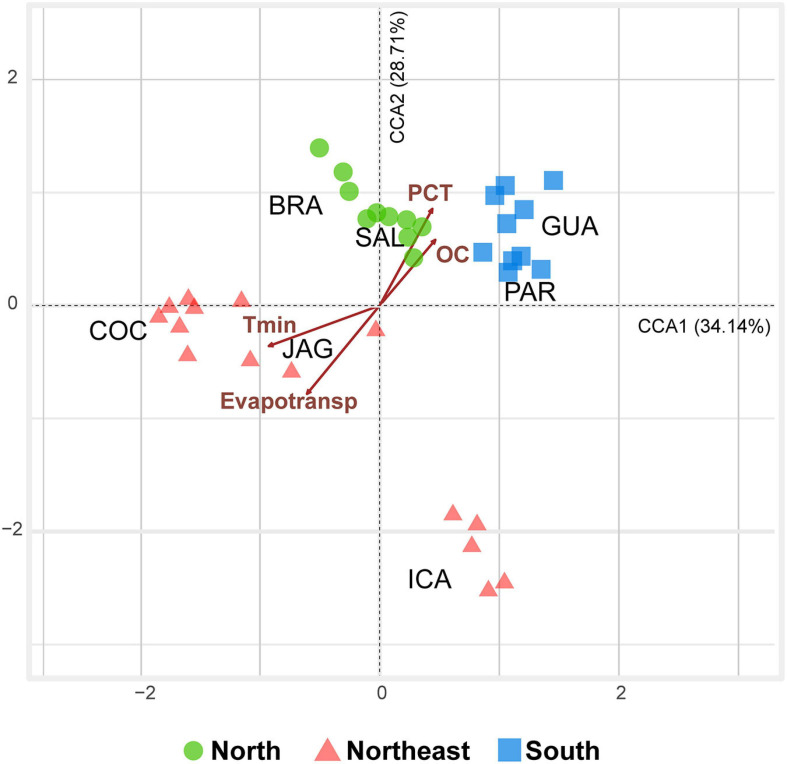
Triplot diagram of the canonical correspondence analysis (CCA) calculated from the dataset containing 35 samples (five sampling points from each mangrove) containing only significant explanatory variables (*P* < 0.05) and prokaryotic ASV composition for samples from the seven studied mangroves [BRA = Bragança (N); SAL = Salinópolis (N); COC = Cocó (NE); JAG = Jaguaribe (NE); ICA = Icapuí (NE); GUA = Guaratuba (S); and PAR = Paranaguá (S)]. CCA 1 (*F* = 3.0563, *P* = 0.001) and CCA 2 (*F* = 2.5695, *P* = 0.001) axes were significant and explained 62.85% of the data. Abbreviated abiotic variables: Tmin (Minimum Temperature); PCT (Precipitation); Evapotrans (Potential Evapotranspiration); and OC (Organic Carbon).

Along the assessed latitudinal transect, minimum temperature, precipitation, organic carbon, and potential evapotranspiration were the main variation drivers of the root-associated microbiome of *R. mangle* (*F* = 2.2378; df = 4; *P* = 0.001). The root-associated communities from northern mangroves were closer to those in the south than those in the northeast. In the ordination, there was possible to separate a group containing samples from northern and southern mangroves, mostly correlated with higher precipitation and organic carbon, as well as lower minimum temperature and potential evapotranspiration. On the other side, northeastern microbiomes were characterized by high potential evapotranspiration and minimum temperature, as well as low precipitation and organic carbon. Axis 1 and 2 explained 62.85% of the dataset variability (Figure 6).

The SIMPER (similarity percentage) analysis displayed that the dissimilarity observed in the ordination was mainly due to variations in the abundance of the orders archaean Marine Benthic Group D and DHVEG, bacteria Desulfobacterales and Campylobacterales, and the genus *Thiofractor*, the latter associated with northern mangroves.

Functional characterization using PICRUSt2 displayed that despite the environmental differences among northern, northeastern, and southern mangroves, in general, they did not differ on predicted functionality (Supplementary Figure 4). Nevertheless, differences in conventional anaerobic ammonium oxidation (Anamox) plus sulfate-reduction with ammonium oxidation (SRAO) and Nitrification, pre-eminent in JAG-NE and COC-NE (northeastern mangroves), should be highlighted (Supplementary Figure 5).

### Network Co-occurrence Patterns

**FIGURE 7:**
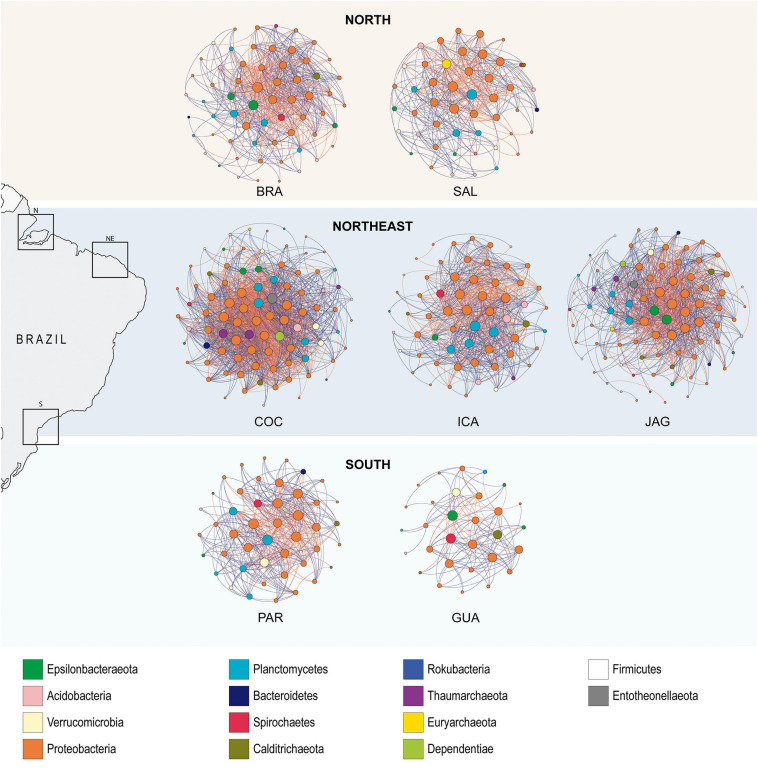
Co-occurrence network based on correlation analysis. A connection stands for a Spearman’s correlation with magnitude >0.7 (positive correlation–blue edges) or <–0.7 (negative correlation–red edges) and statistically significant (*P* < 0.01). The size of each node is proportional to the number of connections (degree). Each node was labeled at the phylum level.

When exploring the complexity of connections within the *R. mangle* root-soil microbiomes, the northeastern mangroves displayed the highest level of complexity and modular structure, whereas the southern mangroves were less complex and displayed the fewest modular networks. Furthermore, southern networks showed lower negative: positive link ratios when compared to northeastern networks (Table 1 and Figure 7).

**TABLE 1:** Topological measures of *Rhizophora mangle* root-zone microbiome co-occurrence networks in seven Brazilian mangroves [BRA = Bragança (N); SAL = Salinópolis (N); COC = Cocó (NE); JAG = Jaguaribe (NE); ICA = Icapuí (NE); GUA = Guaratuba (S); and PAR = Paranaguá (S)].

Network properties	BRA	SAL	COC	JAG	ICA	PAR	GUA
Number of nodes^a^	104	56	95	94	120	91	39
Number of edges^b^	529	369	1,402	958	638	357	131
Positive edges^c^	331	247	791	561	393	237	103
Negative edges^d^	198	122	611	397	245	120	28
Negative: Positive links ratio^e^	0.60	0.49	0.77	0.71	0.62	0.51	0.27
Modularity^f^	1.833	1.280	3.438	2.617	1.673	1.425	0.704
Number of communities^g^	41	9	6	11	6	47	3
Network diameter^h^	6	5	6	6	4	3	4
Mean path length^i^	1.885	1.805	1.714	1.823	1.595	1.562	1.806
Mean degree^j^	7.667	6.589	14.758	10.191	9.815	7.438	3.359
Mean clustering coefficient^k^	0.206	0.301	0.381	0.324	0.348	0.198	0.246

*^*a*^Microbial taxon (at ASV level) with at least one significant (*P* < 0.01) and strong correlation (FastSpar >0.7 or <−0.7).*

*^*b*^Number of connections/correlations obtained by FastSpar analysis.*

*^*c*^FastSpar positive correlation (>0.7 with *P* < 0.01).*

*^*d*^FastSpar negative correlation (<−0.7 with *P* < 0.01).*

*^*e*^Ratio between the number of negative and positive edges.*

*^*f*^The capability of the nodes to form highly connected communities, that is, a structure with high density of between node connections (inferred by Gephi).*

*^*g*^A community is defined as a group of nodes densely connected internally (Gephi).*

*^*h*^The longest distance between nodes in the network, measured in number of edges (Gephi).*

*^*i*^Mean network distance between all pairs of nodes or the mean length of all edges in the network (Gephi).*

*^*j*^The mean number of connections per node in the network, that is, the node connectivity (Gephi).*

*^*k*^How nodes are embedded in their neighborhood and the degree to which they tend to cluster together.*

Co-occurrence networks captured 131 (number of edges in GUA-S) to 1,402 (number of edges in COC-NE) associations among 39 (number of nodes in GUA-S) to 120 (number of nodes in ICA-NE) ASVs. The mean degree (node connectivity) was highest in the northeastern samples (NE mean value = 11.588; N mean value = 7.128; and S mean value = 5.399), indicating higher connectivity among northeastern mangrove populations. GUA-S samples displayed a low mean degree compared with other mangroves, even with PAR-S, indicating a poorly connected community. Besides, modularity, a topology feature that indicates compartmentalization, reached higher values in the northeastern samples, along with BRA-N. GUA-S stood out for the lowest modularity. The mean path length between all pairs of nodes of northeastern mangroves did not differ from the other mangroves, and their diameters were comparable with the mangroves from the north, despite exhibiting higher numbers of nodes and edges. The clustering coefficient, which indicates how nodes are embedded in their neighborhood and, thus, the degree to which they tend to cluster together, was higher in northeastern samples (NE mean value = 0.351; N mean value = 0.254; S mean value = 0.222), indicating highly connected networks.

The diversity of taxa present in each network and their respective importance in the communities’ connections, i.e., their degree, demonstrated that northeastern mangroves networks contained taxa with higher degrees, while the northern and southern networks displayed lower degrees. The number of phyla in each network ranged from 12 (PAR-S and GUA-S) to 20 (COC-NE and SAL-N), but nine phyla were responsible for the highest degrees in these networks, namely Proteobacteria and Epsilonbacteraeota in BRA-N; Planctomycetes, Euryarchaeota and Proteobacteria in SAL-N; Proteobacteria, Entotheonellaeota, and Dependentiae in COC-NE; Proteobacteria and Epsilonbacteraeota in JAG-NE; Planctomycetes and Proteobacteria in ICA-NE and PAR-S; and Epsilonbacteraeota, Spirochaeta, Proteobacteria, Calditrichaeota, and Verrucomicrobia in GUA-S. Some non-dominant phyla proved to be important based on the co-occurrence networks, such as Entotheonellaeota, Dependentiae, Spirochaeta, Calditrichaeota, and Verrucomicrobia.

## Discussion

The soil microbial communities under *R. mangle* root zones were analyzed herein using a metabarcoding approach in three geographical regions: north and south (extremes of distribution), and northeastern Brazil. We demonstrated that precipitation and organic carbon were the strongest selection factors for the north and south, while high evapotranspiration and temperature were the main drivers in the northeast (Figure 6). Accordingly, although we analyzed the root zone of the same species of plant (*R. mangle*), the assessed microbial communities exhibited different richness and compositions. Also, the co-occurrence networks from southern-mangroves were less-complex compared to northeastern ones. This finding may reflect the endurance of microbial communities tailored to deal with the more stressful conditions of semi-aridity in the Brazilian northeast.

Herein, we worked with ASVs, which allows for the discrimination of only one nucleotide in a sequence, resulting in higher taxonomic resolution, which may reveal the distribution of ecotypes across environments and aid in revealing overlooked ecological patterns (Chafee et al., 2018; Garcia-Garcia et al., 2019).

### Microbial Communities From Northeastern Mangroves Displayed Higher Alpha Diversity

Soil microbiomes associated to *R. mangle* of northeastern mangroves were more diverse than the northern and southern ones, even though the southern mangrove PAR-S displayed the highest total number of sequences. This is in line with previous studies that have been showing site specific differences in bacterial richness and composition (Fernandes et al., 2014; Gomes et al., 2014; Basak et al., 2016; Liu et al., 2017; Ullah et al., 2017; Yun et al., 2017; Zhou et al., 2017; Zhu et al., 2018; Tong et al., 2019; Erazo and Bowman, 2021) and indicate that soil or site conditions should be considered along with geographical distance to understand microbial community patterns. Northern and southern mangroves, for instance, displayed more similar microbial diversity than expected considering their geographical distance.

Most datasets were dominated by Proteobacteria, mainly by Deltaproteobacteria and Gammaproteobacteria, which are part of the core microbiome of mangroves worldwide (Dias et al., 2011; Andreote et al., 2012; Zhu et al., 2018; Cotta et al., 2019; Luis et al., 2019; Sadaiappan et al., 2019). Other abundant phyla comprise Planctomycetes, Acidobacteria, Bacteroidetes, and Chloroflexi, in line with the literature on mangrove microbiomes (Marcos et al., 2018; Zhang et al., 2018; Zhu et al., 2018; Cotta et al., 2019; Luis et al., 2019; Sadaiappan et al., 2019; Tong et al., 2019). Desulfobacterales was the most abundant order, as commonly observed in mangrove surface soils (Mendes and Tsai, 2014; Zhang et al., 2018; Zhu et al., 2018; Luis et al., 2019; Sadaiappan et al., 2019), and was highlighted by SIMPER analysis as one of the main responsible for the differences between the studied mangroves. This order comprises anaerobic members involved in sulfur and carbon cycling (Mendes and Tsai, 2014; Zhu et al., 2018) as well as in the transformation of methane and nitrogen, which indicates their importance for mangrove ecosystems (Andreote et al., 2012).

Another important group was Euryarchaeota, especially in ICA-NE and GUA-S. Observing this phylum in such high amounts is a novel finding, since it has been reported in mangroves, but in lower abundances (Andreote et al., 2012; Mendes and Tsai, 2014; Basak et al., 2016). Nevertheless, it is important to highlight that those studies utilized different methodological approaches and primers, which can affect the ratio of retrieved sequences. Euryarchaeota comprises members related to active carbon transformation through methanogenesis, which occurs in the anaerobic sediments found in mangroves (Taketani et al., 2010).

Thermoplasmata class dominated Euryarchaeota sequences from *R. mangle* roots in Florida (United States) mangroves under neutral pH and high sulfur content (Marcos et al., 2018), in pH neutral upper soil layers in a mangrove creek in Cardoso Island (SP, Brazil) (Dias et al., 2011; Otero et al., 2014), and in surface neutral to slightly acidic and rich in iron mangrove soils in New Caledonia (Luis et al., 2019). This preference for acidic and sulfur- or iron-enriched soils may explain the high amounts of Thermoplasmata in GUA-S, but its enrichment in ICA-NE needs to be further investigated (Supplementary Figure 3). Herein, most Euryarchaeota were assigned to the Marine Benthic Group D order (MBG-D), long recognized from 16S rRNA gene surveys in benthic environments (Zhou et al., 2019), and also highlighted by SIMPER analysis.

The phylum Epsilonbacteraeota (phyl. nov.) (Waite et al., 2017), which was noteworthy in northern mangroves, emerged after recent studies reassigned the Epsilonproteobacteria class to a new phylum for constituting a monophyletic unit. Despite possessing autotrophic and thermophilic ancestors, this phylum also comprises heterotrophic and mesophilic members from the Campylobacterales order, involved in carbon and nitrogen fixation, assimilatory nitrate and nitrite reduction, thiosulfate oxidation, and polysulfide reduction (Waite et al., 2017), which explains their detection in mangroves. Also, Campylobacterales was one of the orders highlighted by SIMPER for the effects of its abundance on the ordination.

### Rare Biosphere Comprises Most of the Indicator Genera

The indicator analysis displayed that of the 33 genera selected as indicators, most of them were associated with northeastern mangroves and comprise rare members of the community. The main genera were assigned to representatives from non-common phyla, such as Elusimicrobia and Deinococcus-Thermus, as well as Proteobacteria (*Modicisalibacter*). These genera comprise bacteria capable of reducing nitrate or sulfur compounds, some of them with particular features, such as *Blastocatella* (Elusimicrobia), previously isolated from semi-arid savanna soil; *Truepera* (Deinococcus-Thermus), resistant to ionizing radiation; *Algiphilus* (Proteobacteria), aromatic hydrocarbon-degrading; and *Thiovulum* (Proteobacteria), rapid swimming bacteria. On the other side, southern mangroves were associated with only two indicator genera, which were *Planktotalea* (Proteobacteria) and SEEP-SRB1 (Sulfate-reducing bacteria cluster). The former was previously isolated from seawater or marine organisms from cold regions (Parte et al., 2020).

Rare species can have a preponderant role for local biodiversity and species turnover, being keystone species for regulating the functioning of ecosystems. In addition, rare species that are considered non-relevant under a given environmental condition may become important under changing situations, offering a pool of genetic resources that may be activated under appropriate conditions (Jousset et al., 2017).

### Microbial Community’s Abiotic Drivers Differed Along the Latitudinal Transect

At a global scale, no single biotic or abiotic feature has consistently emerged as the most important determinant of mangrove soil microbial community. Mangrove forests exist in a very broad range of environmental conditions (Kauffman et al., 2020), and the sampled mangrove sites reflected the broad precipitation, salinity, temperature, and evapotranspiration gradients in which they exist and their influence on the associated microbiomes. Traditionally, the main forcing functions over mangrove forests along the Brazilian coast are tides, rainfall, evapotranspiration, and temperature, which vary widely over more than 8,000 Km of coastline (Schaeffer-Novelli et al., 1990). Most factors, such as temperature, rainfall, and evapotranspiration, are related to latitude and have influence on biogeographical distributions (Prosser et al., 2007) with important consequences on the microbial activity at upper soil layers (Gomez et al., 2020).

Many factors have been identified as change drivers concerning the structure and distribution of mangrove microbial communities, but all consisted of local factors, such as the presence/absence of plants and plant species (Jiang et al., 2013; Gomes et al., 2014; Prakash et al., 2015; Chen et al., 2016; Liu et al., 2017; Tong et al., 2019), soil or sediment depth (Taketani et al., 2010; Otero et al., 2014; Basak et al., 2016; Luis et al., 2019), anthropogenic activities (Dias et al., 2011; Fernandes et al., 2014; Nogueira et al., 2015; Erazo and Bowman, 2021), tidal cycles (Zhang et al., 2018), organic carbon and nitrogen (Chen et al., 2016), ammonia nitrogen (Zhu et al., 2018), and silt-clay content (Colares and Melo, 2013). Herein, examining the microbiota below *R. mangle* along a latitudinal transect, regional factors (temperature, precipitation, and evapotranspiration) were identified as the main drivers of the prokaryotic community, with only organic carbon considered a local factor. Another important factor, the tidal cycle, was not analyzed in this study. However, tidal regimen differs widely between the northern, northeastern, and southern coastal systems with a macrotidal regimen in the north, mesotidal regimen in the northeast, and microtidal regimen in the south (Knoppers et al., 1999).

The mangroves from northern and southern Brazil represent extremes in *R. mangle* distribution, but are not extremes in terms of temperature, precipitation, potential evapotranspiration, and organic matter. Although all mangroves grow in fluctuating environmental conditions, such as tidal cycles, high salinity, high temperatures, and anaerobic muddy soils (Jacotot et al., 2019), among the analyzed mangroves, northeastern mangroves are the ones that inhabit the most extreme conditions. Located on the semi-arid coast of Brazil (Fernandez et al., 2019), these mangroves are subjected to the highest minimum temperatures and potential evapotranspiration, as well as the lowest precipitation and organic-matter contents. Based on the physical and chemical variables alone (Figure 2), northern and southern mangroves already displayed a certain degree of similarity, which was reinforced by adding the biological information on the ASVs through CCA ordination (Figure 6). Thus, through the CCA it is possible to display the effect of the biotic interactions on the distribution of the study areas.

The drivers identified in this study strongly influence the ecology and life history of almost all life forms, from macro-organisms to microbes. Changes in temperature and precipitation can lead to variations in mangrove species composition and growth (Alongi, 2008). Indeed, the temperature is one of the main ecological drivers worldwide, and the low annual mean air temperature is one of the main Neotropical mangrove structure and productivity limiters (Ochoa-Gómez et al., 2019). Minimum temperature was one of the main factors for the proximity of northern and southern mangrove microbiomes and the separation from northeastern samples. The semi-arid coast (Northeast Brazil) displays dominant features that help to differentiate it from others, such as water deficit caused by the low rainfall, water impoundment by dams, and high evapotranspiration rates, which contribute to increased saline intrusion and the expansion or landward migration of mangroves (Godoy and Lacerda, 2015).

Precipitation is another pivotal factor, especially important for mangrove trees, which develop better in areas with precipitation above 1,500 mm yr^–1^ and reach their maximum in areas receiving more than 2,500 mm yr^–1^ (Schaeffer-Novelli et al., 1990). Nevertheless, southern mangroves are usually composed of shorter trees and less structurally complex mangrove swamps (Lana et al., 2001), due to lower sea surface temperature (Ximenes et al., 2018). Our data indicate that the microbial community responded similarly, as samples from the south (PAR-S and GUA-S) consisted in less diverse and complex microbial communities.

### Northeast Mangroves Are Inhabited by Rich and Complex Microbial Communities

Herein, we started from a hypothesis that northern and northeastern mangroves would exhibit more similar microbial communities as *R. mangle* propagules dispersion had originated in the north and was constrained by temperature decreases southward in South America (Francisco et al., 2018), which resulted in genetic divergence between north and south *R. mangle* populations. However, we observed a greater similarity between northern and southern red-mangrove microbiomes and higher diversity in northeastern mangroves.

It is well known that the diversity of the plant community and the genotypes of individual plants can influence the composition of associated microbial communities. This happens because microorganisms that live in the rhizosphere are attracted by and feed on rhizodeposits, such as nutrients, exudates, border cells, and mucilage released by the plant root, factors related to the plant genotype. Also, differences in root architecture as well as in the amount and type of rhizodeposits influence greatly the composition of associated microbiota (Craig et al., 2020). Another factor to be considered is plant physiological activity as specific metabolites released into the rhizosphere can trigger multiple responses by different soil microorganisms, such as germination, branching, quorum sensing, or metabolization, which will lead to the establishment of symbiosis or to ward off pathogens and pests (Philippot et al., 2013). *R. mangle* populations from the South of Brazil have been previously reported for showing a low genetic diversity (Francisco et al., 2018), which is alarming considering the structural role *Rhizophora* spp. have in the species-pool of Neotropical (AEP) mangroves. *R. mangle* populations from the South of Brazil were reported to possess insignificant heterozygosity related to dispersion limitation (Francisco et al., 2018), a process that can also affect associated microbial community assembly. However, these southern populations possess an important role in the southward expansion of mangrove forests in the face of climate change and this low genetic diversity can result in less chance to have the necessary traits to deal with environmental changes. When we combine such low genetic diversity with the less diverse and complex associated soil microbial communities observed herein, we have indicators that the ecosystem biodiversity and functioning may be threatened. Soil microbial diversity is usually not considered in conservation efforts, although they are key to soil functionality and provide many important ecosystem functions and services, such as biogeochemical cycling, plant growth, and carbon sequestration for and beyond the whole ecosystem (Dubey et al., 2019).

Reduced microbial diversity and less complex networks, observed in the southern mangroves, are thought to lead to lower levels of soil functioning due to fewer taxa to support functional redundancy and/or different functions (Wagg et al., 2019). However, when analyzing data from PICRUSt2, we did not observe functional differences among all studied mangroves, which can indicate that functional redundancy is still maintained. Nevertheless, one could question if this is enough to guarantee ecosystem resistance to ongoing and future environmental changes. Considering the limitations of this approach, which is based on potential or predicted pathways, and the importance of southern mangroves in the southward migration of mangroves, more attention should be dedicated to this aspect using functional approaches, such as metatranscriptomics and metabolomics.

When we change the focus to mangroves of the Northeast, we have an opposite situation in which, possibly, the harsh environmental conditions of the semi-arid coast have made unique niches available, in which microbial populations interact differently from their northern and southern counterparts. In the northeast, only ICA-NE differed from the other northeastern samples. This could be due to its lagoon estuarine system, in which a channel directs waters from a coastal lagoon and aquifers toward the mangrove throughout the year (Meireles et al., 2017), maintaining freshwater input despite low rainfall. This system does not operate in the other two northeastern mangroves, which may indicate water deficit as a strong selection force for microbial communities. Nevertheless, as with its northeastern counterparts, ICA-NE is subject to regional climatic factors, such as low rainfall as well as elevated temperature, evapotranspiration, and salinity, which could explain its difference from northern and southern mangroves.

Despite rainfall decreases may limit seed germination due to higher soil salinities (McKee et al., 2012), mangroves in northeastern Brazil appear to be expanding landward, in the opposite direction to the observed global trend (Godoy and Lacerda, 2015). Considering the important role soil microbiomes play in nutrient cycling and plant fitness, detecting more-complex microbial networks in northeastern samples suggests that microbes may play a role in the resistance and successful expansion of such mangroves, which should be further investigated.

Thus, the divergence of northeastern mangroves was confirmed by microbial co-occurrence networks, which revealed more complex and intricate networks in northeastern microbiomes and very simplified networks in southern samples. Beyond the information provided by standard taxonomic approaches and how environmental properties shape microbial communities, co-occurrence networks provide insights on community stability, ecosystem functioning, and biotic factors (Barberán et al., 2012). Northeastern networks display highly connected ASVs, structured among densely connected groups of nodes, forming a clustering topology. Modularity (indicative of compartmentalization) reached higher levels in the northeastern samples.

### Biotic Interactions Respond to Stress or Perturbation Gradient

The study of connectivity is essential for providing recruitment sources for the whole community. Also, the ratios of negative:positive association among taxa were lower in samples of the south, which are indicative of lower stability in those communities (Hernandez et al., 2021), in accordance with the Stress Gradient Hypothesis. According to this hypothesis, the frequency of competitive interactions (negative edges in the network) decreases with the intensification of stress. On the other side, facilitative interactions (positive edges) increases along with stress increases. Positive relationships, such as mutualism, represent higher niche overlap, while negative relationships, such as parasitism and competition, indicate divergent niches. As environmental stress increases, there can be a substitution of competitive taxa by slow-growing stress-tolerant species (Bertness and Callaway, 1994). Also, when considering the community stability to disturbances, positive connections can destabilize microbial communities by positive feedback loops in which decreases in one taxon led to decreases also in reliant taxa. More stable communities have more limited shifts in composition in response to environmental perturbations and/or are more likely to return to their equilibrium after a perturbation (Hernandez et al., 2021).

On the other hand, network co-occurrence analyses of microbial communities from other soil ecosystems have reported increasing complexity related to more-disturbed areas, such as Cd-contaminated soils (Wang et al., 2019), arid soil under grazing (Marcos et al., 2019), PAH-contaminated riverine sediments (Yan et al., 2019), and soils undergoing nutrient loss (Liu et al., 2019). This greater co-occurrence pattern can be explained by modifications in prevalent conditions that remove limits on other taxa and introduce additional niches, similar to that proposed by the Intermediate Disturbance Theory (Padisak et al., 1993). According to this theory, higher diversity is reached at an intermediate frequency or intensity of disturbances. Thus, mangrove soil bacteria in the northeast may form more-complex networks as a result of disturbances caused by water deficit. Protection status also corroborates in demonstrating how exposed northeastern mangroves are to disturbance, as only 23.09% of them are located in coastal protected areas, while 87.98 and 69.38% are protected in northern and southern mangroves areas, respectively. In addition to this lower degree of legal protection, northeastern Brazil mangroves are threatened by extensive shrimp farming, the greatest cause of mangrove loss worldwide (Pelage et al., 2019).

Historical data indicate that mangrove forests are considerably resilient, displaying a significant ability to adapt to changing conditions (Alongi, 2008). The northeastern mangroves assessed herein, subject to high temperatures and low rainfall, seem to possess an associated microbiota adapted to respond to these harsh conditions, as demonstrated by the present results, i.e., high diversity and more-complex networks. Moreover, while some changes can lead to mangrove mortality and loss, others can lead to mangrove expansion. Mangroves are expected to be sensitive to hypersalinity, most prevalent in arid and semi-arid areas (Lovelock et al., 2016), such as northeastern Brazil. Nevertheless, the mangroves in this region display a trend toward landward expansion, related to low rainfall and consequent saline intrusion (Godoy et al., 2018). This trend along with the southward expansion at the southern limit are adaptations to climate change (Godoy and Lacerda, 2015). For instance, Cocó (COC-NE) and Jaguaribe (JAG-NE) mangroves have expanded, respectively, 22.8 and 4.1% in the last 20 years (Godoy et al., 2018). Considering the differences observed in the northeastern microbial communities, one might consider what role they have in this process. Although ecologists have long known that precipitation and salinity regimes govern the global distribution, abundance, and species richness of mangrove forests (Spalding et al., 2010), the microbial communities in northeastern Brazil were shown to be adapted and possibly responsible for mediating the persistence and expansion of mangrove forests.

Rainfall variability and droughts are among the most important harbingers of climate change for mangroves (Sippo et al., 2018). Reductions in freshwater supply induce hydrological droughts, reinforcing evapotranspiration effects and increasing water and sediment salinity. These factors are thought to weaken the competitive ability of mangroves relative to adjacent communities, reducing their spatial area, productivity, and health. Reduced precipitation may be responsible for 11% of the global reduction in the spatial extent of mangroves (Mafi-Gholami et al., 2019). Mangroves from the semi-arid coast of Brazil have historically experienced these conditions and have adapted to persist in such harsh circumstances. Our study provides support for the role of microbes in this adaptability.

On the other hand, cold southern temperatures, which can lead to mangrove mortality, loss of above-ground biomass, lower productivity, and decreased reproduction (Lovelock et al., 2016), also affect their less diverse and complex root-associated microbial communities. These findings are important, as a positive relationship between biodiversity and marine ecosystems functioning is noted, which means that biodiversity loss could result in impaired microbial ecosystem functioning through reduced functional redundancy and interaction networks (Konopka et al., 2015). This is particularly important considering the poleward shifts in mangrove distributions expected to occur due to global climate change (Whitt et al., 2020). Here, semi-arid mangroves stand out as important pools of microbial diversity and complexity, which are pivotal to mangrove adaptation in the Anthropocene.

## Conclusion

Considering the findings reported herein, it seems that northeastern and southern Brazilian mangroves, as important centers of mangrove dispersion in the Anthropocene, are going in opposite directions. While we are concerned with global warming, the colder temperatures of the south are limiting mangrove-associated microbial communities’ richness and complexity while elevated temperatures and evapotranspiration, as well as reduced precipitation, lead to saline intrusion and the harboring of a diverse and complex pool of microorganisms in the northeast.

Microorganisms could be used for ecosystem recovery from anthropogenic disturbances or assisted ecological restoration based on the role of microbial diversity in maintaining the dynamic balance and functional equilibrium essential for mangrove sustainability. Considering the differences among the bacterial profiles of the mangroves studied herein, the impact on or of this unseen diversity should be addressed in ecosystem management as well as in the development of evidence-based microbial interventions and the exploration of biotechnological tools. The adaptive power provided by microorganisms may be the answer to adaptability in our changing world.

## Data Availability Statement

The datasets presented in this study can be found in online repositories. The names of the repository/repositories and accession number(s) can be found below: https://www.ncbi.nlm.nih.gov/bioproject/PRJNA283936.

## Author Contributions

WB and VM contributed to the conception and design of the study and acquired the samples and data. WB, LN, and TT performed the data analysis and interpretation. TT, AR, and VM wrote the first draft of the manuscript. All authors contributed to the manuscript revision, read, and approved the submitted version.

## Conflict of Interest

The authors declare that the research was conducted in the absence of any commercial or financial relationships that could be construed as a potential conflict of interest.

## Publisher’s Note

All claims expressed in this article are solely those of the authors and do not necessarily represent those of their affiliated organizations, or those of the publisher, the editors and the reviewers. Any product that may be evaluated in this article, or claim that may be made by its manufacturer, is not guaranteed or endorsed by the publisher.

## Footnotes

1
https://www.ncbi.nlm.nih.gov/bioproject/PRJNA283936
