# Supplementary material for: Brazilian Semi-Arid Mangroves-Associated Microbiome as Pools of Richness and Complexity in a Changing World
Source: Front Microbiol. 2021 Aug 26;12:715991. doi: 10.3389/fmicb.2021.715991 (PMC8427804; doi:10.3389/fmicb.2021.715991)
Supplement: Supplementary file 1 [file Data_Sheet_1.PDF]

Supplementary information for the manuscript “Brazilian semi-arid mangroves-associated microbiome as pools of richness and complexity in a changing world” by Tavares et al.

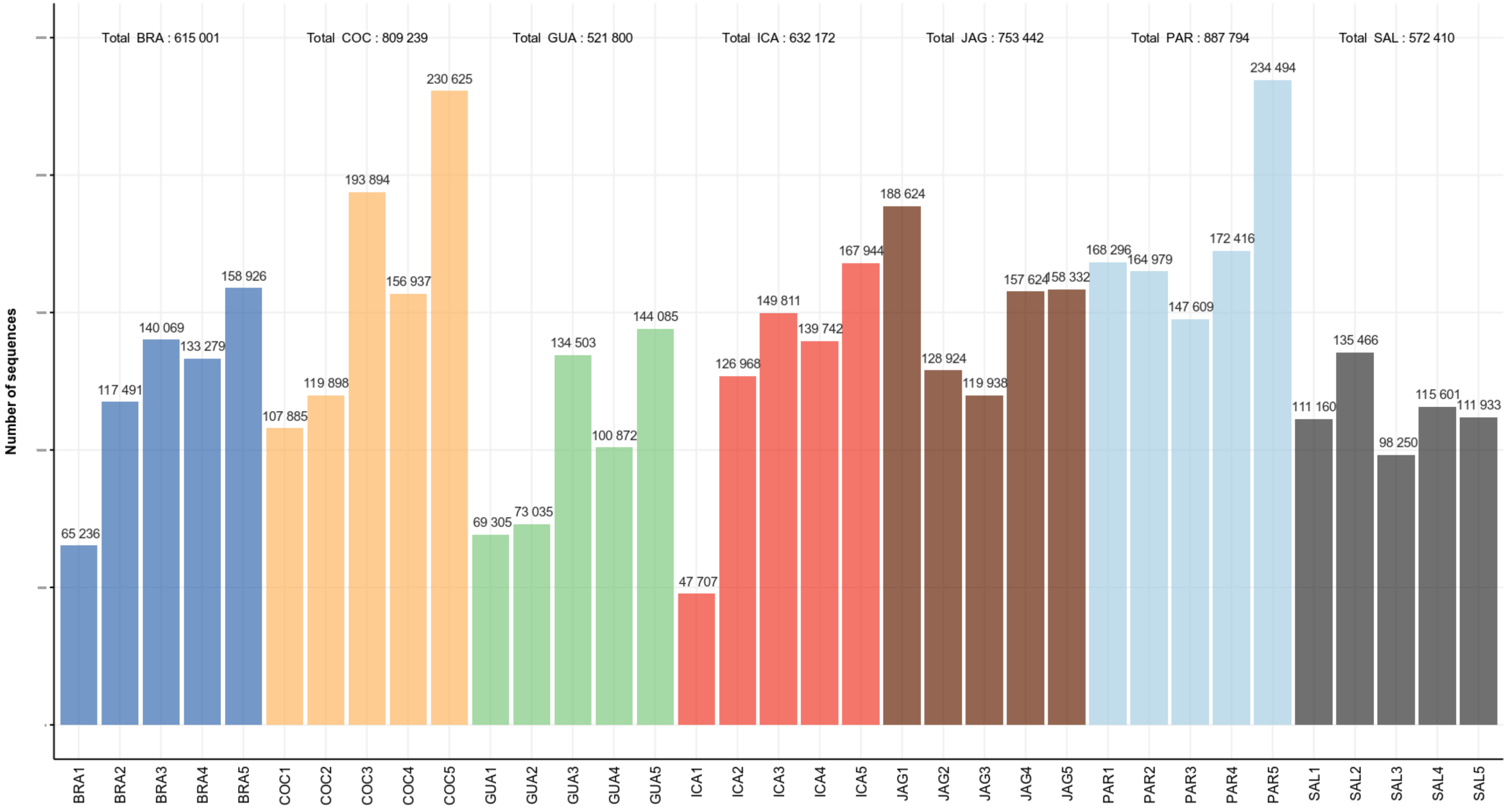


Supplementary Figure 1 - Total number of sequences in each sampling point after quality filtering and removal of chimeric samples, Mitochondria, Chloroplast, Non-assigned and Eukarya reads. The figure displays each of the five sampling points for each mangrove and the total number of sequences per mangrove is shown in the upper zone of the figure. BRA = Bragança (N); SAL = Salinópolis (N); COC = Cocó (NE); JAG = Jaguaribe (NE); ICA = Icapuí (NE); GUA = Guaratuba (S); and PAR = Paranaguá (S).

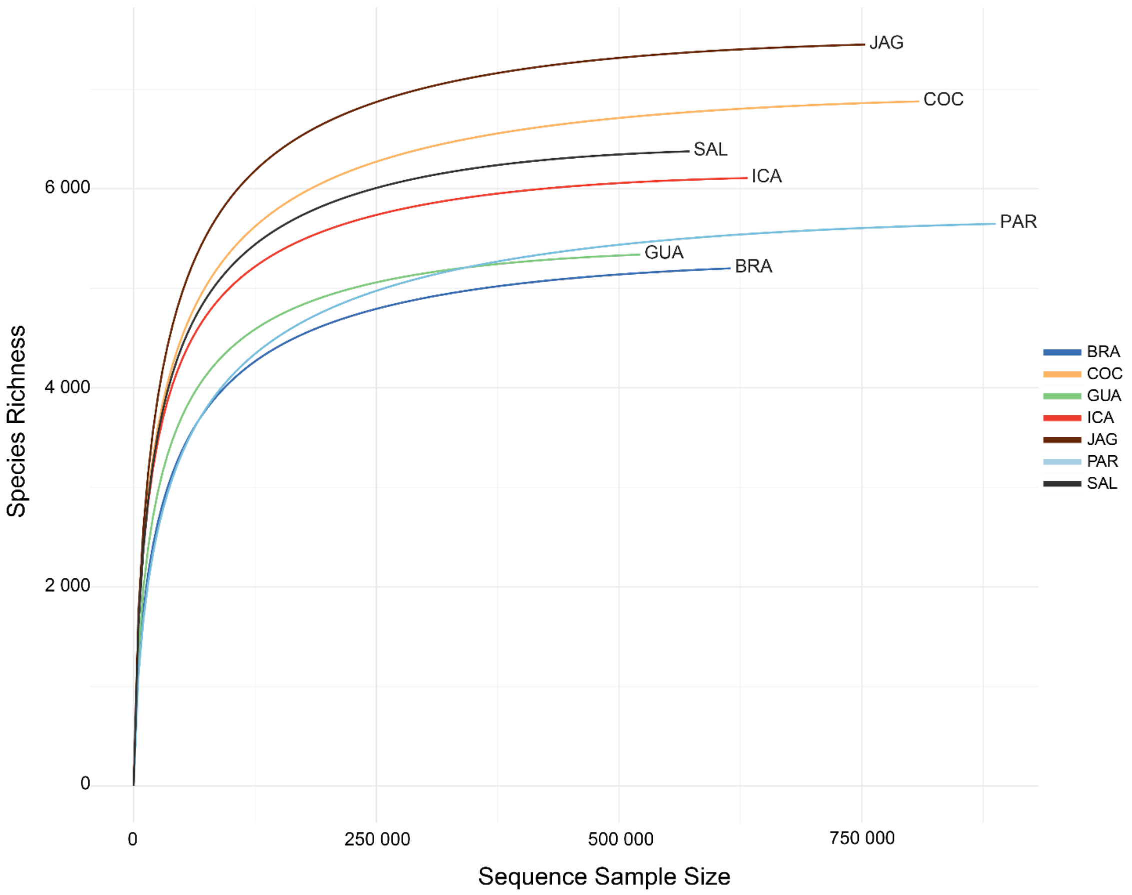


Supplementary Figure 2 – Rarefaction curves generated from the species richness for the seven studied mangroves. The X-axis presents the number of sequences in each mangrove, while the Y-axis shows the number of species (BRA = Bragança (N); SAL = Salinópolis (N); COC = Cocó (NE); JAG = Jaguaribe (NE); ICA = Icapuí (NE); GUA = Guaratuba (S); and PAR = Paranaguá (S).

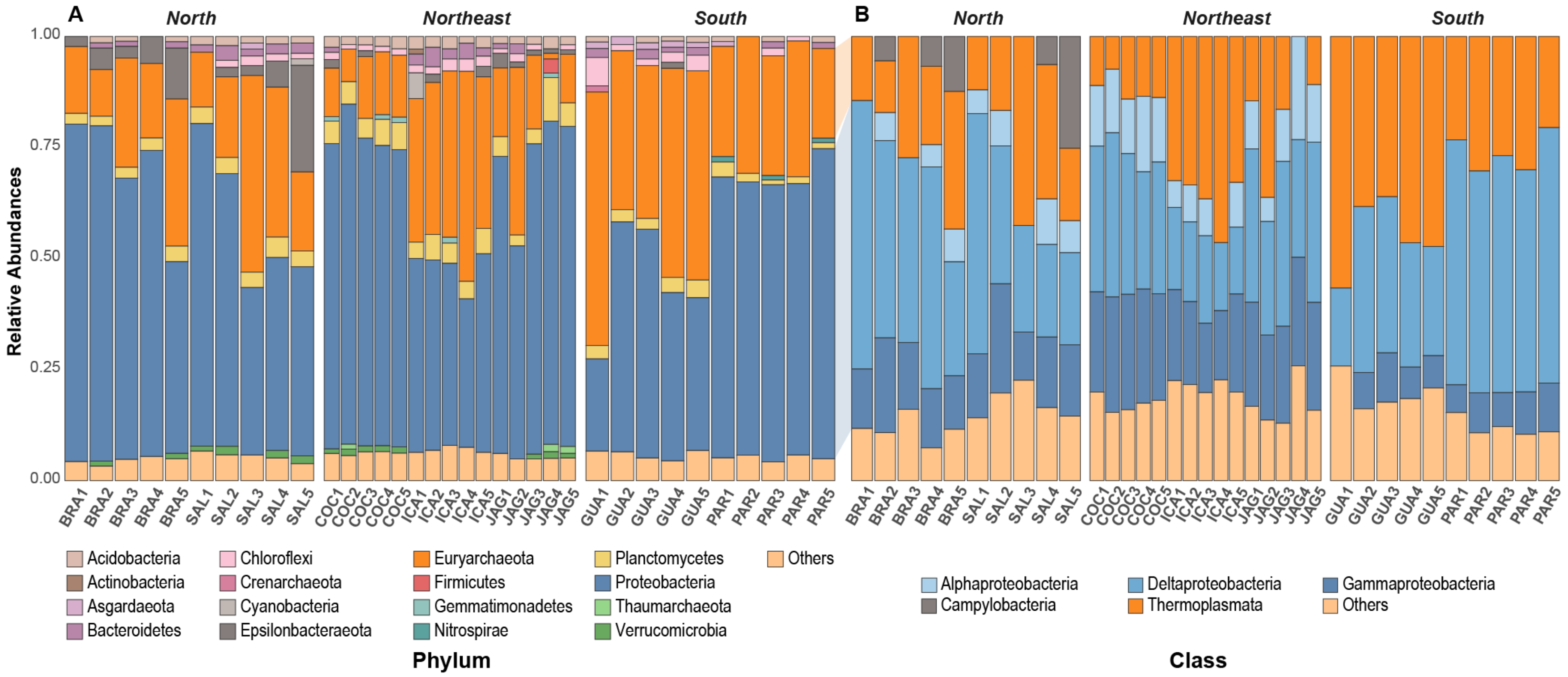


Supplementary Figure 3 – Comparison of taxonomic distributions of Bacteria and Archaea at the phylum (A) and class (B) level in *Rhizophora mangle* root-associated soil. Stacked bar plot of the relative abundance above 0.01 for each sampling point (summed from three replicates each). Phyla and classes with relative abundance < 0.01 are displayed as "Others".

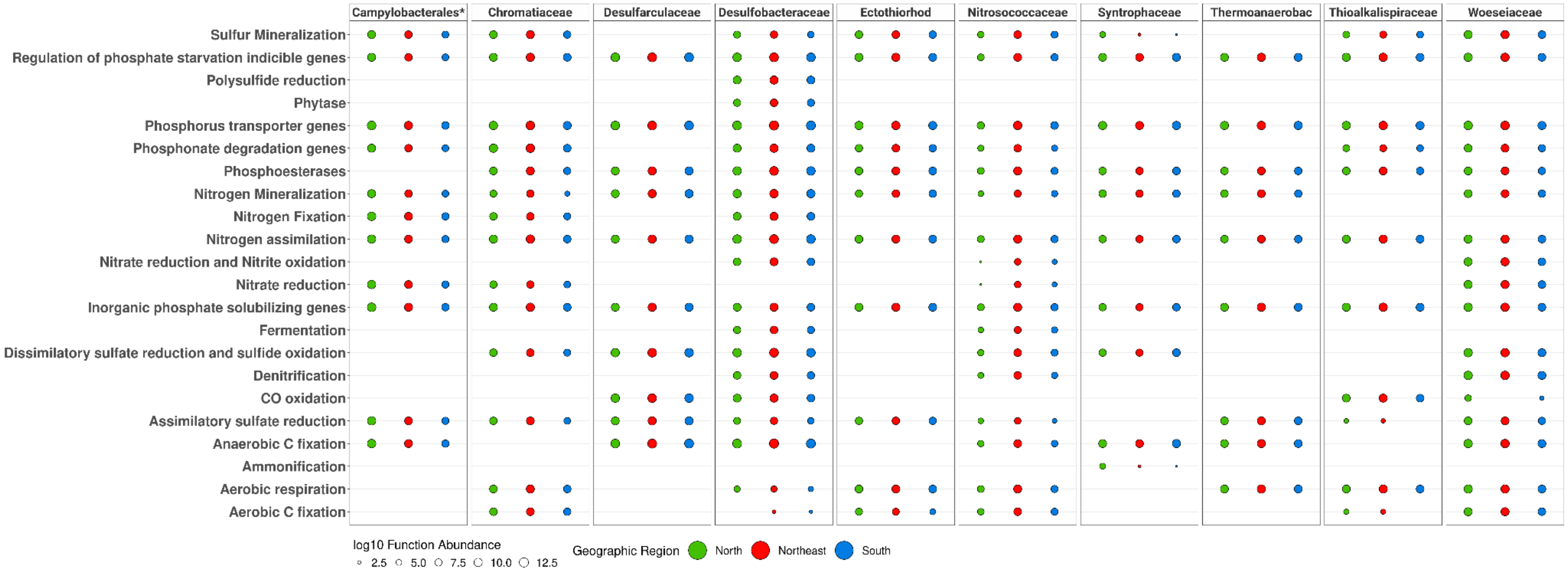


Supplementary Figure 4 – Main microbial contributors to KEGG Orthologs associated with Carbon, Nitrogen, Sulphur, and Phosphorus cycles based on PICRUSt2 analysis generated by using the 16S rDNA data on *Rhizophora mangle* root-associated soil microbiomes. In the figure, "Ectothiorhod" refers to Ectothiorhodospiraceae, "Thermoanaerobac" refers to Thermoanaerobaculaceae, and "Campylobacterales*" refers to Campylobacterales Incertae Sedis.

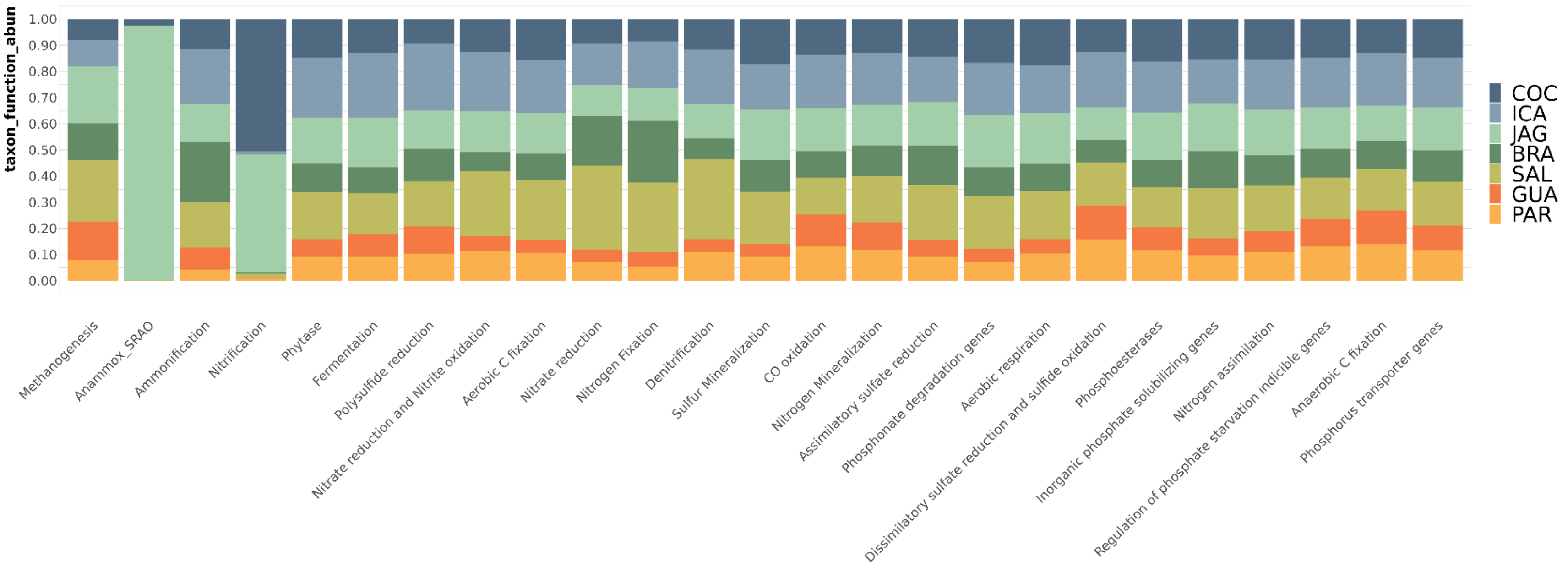


Supplementary Figure 5 – PICRUSt2 analysis of predicted KEGG Orthologs associated with Carbon, Nitrogen, Sulphur, and Phosphorus cycles generated by using the 16S rDNA data on mangrove-associated microbiomes.

Supplementary Table 1 – Counts of raw sequencing reads during quality processing and total number of filtered reads (nonchim). Five sampling points for each mangrove were sampled, with triplicates subsamples for each, totaling 105 samples (five samples with three subsamples per mangrove). Input represents the total resulting number of raw sequences; filtered represents the total number of samples after quality filtering; and nonchim represent the total number of non-chimeric samples.

**Supplemented as Excel file.**

Supplementary Table 2 – General characterization of the datasets and alpha diversity. No. seqs = number of sequences; No. sing = number of singletons; Good's = Goods coverage; S obs = observed richness.

| Mangroves | No. seqs | No. sing | Good's | Inverse Simpson | Shannon | S obs | Chao1 |
|---|---|---|---|---|---|---|---|
| **Bragança (N)** | 615 001 | 243 | 99.96 | 28.83 | 5.74 | 5 200 | 5274.82 |
| **Salinópolis (N)** | 572 410 | 174 | 99.97 | 75.43 | 6.64 | 6 375 | 6405.78 |
| **Cocó (NE)** | 809 239 | 209 | 99.97 | 140.44 | 6.95 | 6 877 | 6928.75 |
| **Jaguaribe (NE)** | 753 442 | 195 | 99.97 | 76.67 | 6.85 | 7 448 | 7490.79 |
| **Icapuí (NE)** | 632 172 | 138 | 99.98 | 233.64 | 6.94 | 6 107 | 6129.56 |
| **Guaratuba (S)** | 521 800 | 202 | 99.96 | 86.56 | 6.22 | 5 338 | 5391.99 |
| **Paranaguá (S)** | 887 794 | 198 | 99.98 | 14.10 | 5.31 | 5 647 | 5693.44 |

Supplementary Table 3 – List of the 33 significant indicator genera ($P < 0.05$) for *Rhizophora mangle* root-associated soil microbiomes and their respective values for North, Northeast, and South regions. The values were obtained by using the function *multipatt* from *indicspecies* package in R.

| **Indicator genus** | **stat** | ***P*-value** | **Indicator value** |
|---|---|---|---|
| **North** | | | |
| *Sulfurovum* | 0.921 | 0.036 | 0.8482 |
| *Blastocatella* | 0.914 | 0.025 | 0.8361 |
| *Thiofractor* | 0.88 | 0.009 | 0.7745 |
| *Truepera* | 0.868 | 0.009 | 0.7536 |
| *Algiphilus* | 0.864 | 0.036 | 0.7459 |
| *Thiovulum* | 0.825 | 0.02 | 0.6804 |
| *Thermotomaculum* | 0.774 | 0.037 | 0.5988 |
| *Altererythrobacter* | 0.727 | 0.009 | 0.528 |
| *Verrucomicrobia 01 ** | 0.696 | 0.04 | 0.4848 |
| **Northeast** | | | |
| *Pontibacillus* | 1 | 0.022 | 1 |
| *Halomonas* | 1 | 0.022 | 1 |
| *Magnetovibrio* | 1 | 0.022 | 1 |
| *Roseimarinus* | 1 | 0.022 | 1 |
| *Modicisalibacter* | 0.99 | 0.022 | 0.9801 |
| *Candidatus_Babela* | 0.979 | 0.022 | 0.9575 |
| *Fulvivirga* | 0.971 | 0.022 | 0.9437 |
| *Bradymonas* | 0.966 | 0.022 | 0.9335 |
| *Halobacillus* | 0.944 | 0.022 | 0.8912 |
| *Tepidibacter* | 0.936 | 0.022 | 0.8767 |
| *X3PJM14* | 0.923 | 0.022 | 0.8518 |
| *Tistlia* | 0.918 | 0.022 | 0.8433 |
| *Halovulum* | 0.912 | 0.022 | 0.8317 |
| *Hypnocyclicus* | 0.91 | 0.022 | 0.8286 |
| *Reinekea* | 0.904 | 0.022 | 0.8174 |
| *Salinirepens* | 0.901 | 0.022 | 0.8114 |

| | | | |
|---|---|---|---|
| *Bythopirellula* | 0.883 | 0.022 | 0.779 |
| *OM60.NOR5. clade* | 0.881 | 0.022 | 0.7753 |
| *Orenia* | 0.856 | 0.022 | 0.7333 |
| *Aquimixticola* | 0.849 | 0.033 | 0.7214 |
| *Pelagibius* | 0.848 | 0.022 | 0.7191 |
| *Vulgatibacter* | 0.8 | 0.022 | 0.6397 |
| **South** | | | |
| *Planktotalea* | 0.825 | 0.036 | 0.68 |
| SEEP.SRB1 | 0.67 | 0.045 | 0.4482 |
